# Acute phase response in lame crossbred dairy cattle

**DOI:** 10.14202/vetworld.2016.1204-1208

**Published:** 2016-11-08

**Authors:** A. Bagga, Swaran Singh Randhawa, S. Sharma, B. K. Bansal

**Affiliations:** 1 Department of Animal Husbandry, CVH, Mehatpur, Jalandhar, Punjab, India; 2 Department of Veterinary Medicine, College of Veterinary Science, Guru Angad Dev Veterinary and Animal Sciences University, Ludhiana - 141 004, Punjab, India

**Keywords:** acute phase proteins, cattle, lameness

## Abstract

**Aim:**

The study was undertaken to study acute phase response based on acute phase proteins (APPs) such as C-reactive protein (CRP), haptoglobin (Hp), serum amyloid A (SAA), and fibrinogen in lame crossbred dairy cattle.

**Materials and Methods:**

Lame animals (n=30) were selected within 3-7 days of being noticed as lame by the farm veterinarian, from a local dairy farm in southeast Ludhiana over a period of 6 months, stratified proportionately with respect to stage of lactation with non-lame healthy cows (n=10). All the cows were otherwise healthy and did not have any other inflammatory problems such as pneumonia, enteritis, mastitis, or any kind of acute uterine inflammation. Blood samples were collected from all the animals; serum and plasma samples were separated and stored at −20°C. The levels of CRP, Hp, and SAA were estimated using Sandwich ELISA, whereas fibrinogen was estimated by heat precipitation method.

**Results:**

SAA levels in lame cows were significantly higher (22.19±0.85 µg/ml), approximately 3 times as compared to non-lame cows (8.89±0.72 µg/ml), whereas serum Hp concentration was approximately 20 times higher in the lame cattle (21.71±3.32 mg/dl) as compared to non-lame cows (1.17±0.07 mg/dl). Fibrinogen also increased in the lame cattle (3.97±0.22 g/L) as compared to non-lame group (1.40±0.17 g/L). Serum CRP levels analyzed in the lame cattle for the first time in the present study, and significant high concentration was appreciated in lame cattle (4.41±0.33 mg/L) as compared to non-lame cattle (0.61±0.14 mg/L). Lame cattle were having more of sole hemorrhages, sole ulcers, and white line lesions as compared to non-lame cattle.

**Conclusion:**

It can be concluded that lame cattle exhibit high levels of APPs including CRP, Hp, SAA, and fibrinogen as compared to non-lame cattle.

## Introduction

Acute phase proteins (APPs) are the blood proteins that change in concentration in response to various inflammatory as well as non-inflammatory conditions in animals [1-7]. APPs are released from the liver after activation of hepatocytes by the proinflammatory cytokines (interleukin 1 [IL-1], IL-6, and tumor necrosis factor-α) which are secreted by the monocytes in response to bacterial toxins or tissue injury. There are basically two types of APPs, negative and positive APPs which increase and decrease in levels in response to any challenge. Albumin and transferrin are the negative APPs, whereas C-reactive protein (CRP), haptoglobin (Hp), serum amyloid A (SAA), and fibrinogen are the positive APPs [8]. Among these, SAA and CRP having molecular weight of 180 and 115 kDa, respectively, and are the first phase APPs which rise as early as 4 h of initiation of inflammation, peak within 1-3 days and then return to normal [9]. In cattle, there is local synthesis of SAA in the udder known as “Milk-SAA” [10]. The Hp (α2 globulin) and fibrinogen with molecular weight 125 kDa and 340 kDa, respectively, are the second-phase APPs which increase 1-3 days after the initiation of inflammation, peak within 7-10 days, and return to baseline values within 2 or more weeks.

APPs, besides inflammatory conditions, have been reported to be very useful for analyzing non-inflammatory conditions such as metabolic disease, pregnancy, parturition, and stress [8]; and APPs are much more useful than classical hematological tests, *viz*., leukocyte count, neutrophil percentage, or immature neutrophil percentage to differentiate between an acute and a chronic state in cattle [11]. Hp is considered better than all other APPs for discrimination between the healthy and diseased calves in a dairy herd. SAA can be regarded as a less suitable indicator of health problem in calves because it is more sensitive and can be increased by a mild stress. SAA and Hp are the major APPs in cattle which increases 10-100 folds, whereas CRP and fibrinogen are the moderate APPs which increase 2-10 fold in case of any external challenge [8]. Hp and SAA have been observed to be of diagnostic value in the lame dairy cows [12]. In another study [13], systemic acute phase response and elevated serum Hp were observed in claw lameness in dairy cattle. SAA and Hp levels have also been observed to increase in lame cattle in response to bacterial infection due to *Fusobacterium necrophorum* [14].

The present study was conducted to analyze various APPs in the cattle suffering from acute foot lameness.

## Materials and Methods

### Ethical approval

Approval was given by the Research Advisory Committee, and samples were collected as per standard sample collection procedure without harming or giving stress to any animals.

### Farm and management

The study was carried on local dairy farm with 800 cows in village Latala on the southeast of Ludhiana, Punjab, from February to July 2014. The animals were housed in a loose housing system. Milking of the animals was done twice daily, and a total mixed ration was fed to the animals according to national recommendations with *ad libitum* access to water. Routine claw trimming was undertaken 6 monthly in all the animals. Cows were observed daily for health status including lameness by the farm veterinarian. Cows found lame were given veterinary treatment as per requirement.

### Animals

Thirty lactating crossbred (HF-Sahiwal) clinical lame cows (lameness score 2 and 3 on numerical rating scale of 0-4) were selected within 3-7 days of being noticed lame by the farm veterinarian, during weekly visits to the dairy farm. Sole ulcers and white line fissures were observed as lesions responsible for clinical lameness in these cows in a previous study [15], so it was supposed that all the clinical lame animals in the present study were having either sole ulcers or white line fissures. All 30 cows were in good general health and did not have any other inflammatory problems such as pneumonia, enteritis, mastitis, or any kinds of acute uterine inflammation. Another 10 non-lame healthy cows (lameness score 0 or no gait abnormality on numerical rating scale of 0-4) were chosen from the same herd as control animals to measure their status of APPs. About 2 ml of blood from each animal was collected, from jugular vein into vials containing clot activator for the collection of serum for analysis of SAA, CRP, and Hp, and another 2 ml was collected in ethylenediaminetetraacetic acid vials for the separation of plasma for fibrinogen estimation. Both serum and plasma were separated using the centrifugation method and stored at −20° for further use.

### Hoof trimming

A total of 13 lactating lame dairy cows and all the non-lame healthy cows were subjected to diagnostic hoof trimming by removing 1 mm of silver from the solar surface after restraining them in the lameness chute and various claw lesions were noticed. Average lesion score was computed for the lame as well as non-lame animals.

### Estimation of APPs

The estimation of CRP and SAA was done by Sandwich ELISA Kits provided by USCN Inc., and the Hp estimation was done by Sandwich ELISA kit provided by Alpco Inc., as per manufacturer guidelines. For the estimation of fibrinogen, refractometer method was used.

### Statistical analysis

The data were analyzed and evaluated statistically using Student’s t-test (independent samples test) using SPSS version 16 software to analyze change in the concentration of the APPs with lameness.

## Results

**Figure-1:**
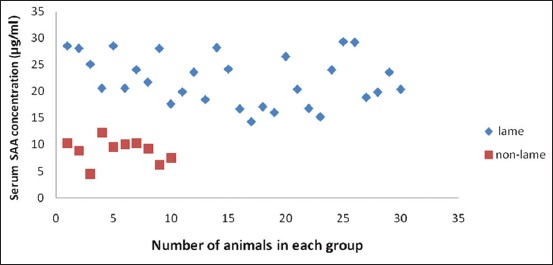
Scatter diagram showing serum amyloid A concentration in lame and non-lame healthy crossbred cows.

**Figure-2:**
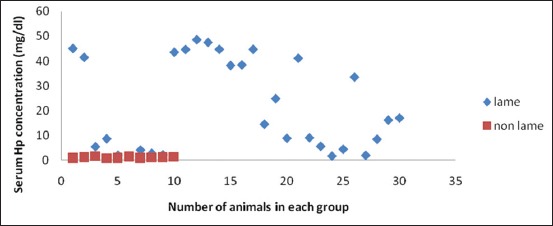
Scatter diagram showing haptoglobin concentration in lame and non-lame healthy crossbred cows.

**Figure-3:**
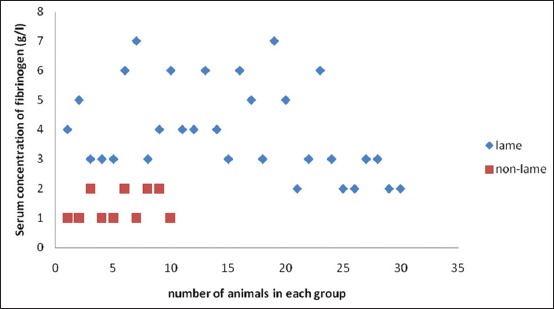
Scatter diagram showing fibrinogen concentration in lame and non-lame healthy crossbred cows.

**Figure-4:**
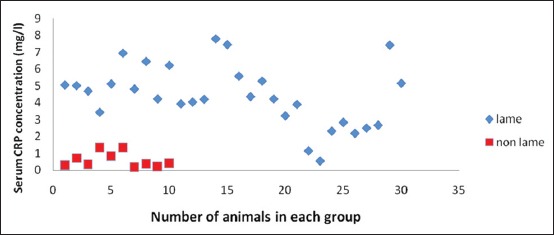
Scatter diagram showing C-reactive protein concentration in lame and non-lame healthy crossbred cows.

Descriptive statistics and results for comparison of lame and healthy crossbred cows are presented in Table-1. It was found that SAA levels in lame cows were significantly high, approximately 3 times (22.19±0.85 µg/ml) as compared to healthy cows (8.89±0.72 µg/ml), whereas serum Hp concentration was approximately 20 times in the lame cattle (21.71±3.32 mg/dl) as compared to non-lame cows (1.17±0.07 mg/dl). Fibrinogen was also high in the lameness group (3.97±0.22 g/L) as compared to control group (1.40±0.17 g/L). Serum CRP levels analyzed in the lame cattle for the first time in the present study were also significantly high in lame cattle (4.41±0.33 mg/L) as compared to non-lame cattle (0.61±0.14 mg/L). All the lame cattle in the present study were having SAA concentration above 14 μg/ml (Figure-1); however, Hp levels were approximately 30 times only in 12 animals with rest of the animals having Hp levels above 1.75 and below 25 mg/dl (Figure-2). There is a 2-3 fold increase in fibrinogen concentration in lame animals (Figure-3). All the lame animals except two in the present study exhibited CRP levels above 2 mg/L indicating its relevance in the diagnosis of inflammatory conditions (Figure-4).

**Table 1:** Acute phase response in lame and non-lame healthy cattle.

APPs	(Mean±SE)	

	Lame cows	Non-lame healthy cows
CRP (mg/L)	4.41*±0.33 (0.55-7.77)	0.61±0.14 (0.19-1.35)
Hp (mg/dl)	21.71*±3.32 (1.75-48.74)	1.17±0.07 (0.90-1.55)
Fibrinogen (g/L)	3.97*±0.22 (2.0-7.0)	1.40±0.17 (1.0-2.0)
SAA (µg/ml)	22.19*±0.85 (14.34-29.3)	8.89±0.717 (4.46-12.30)

* Significant at p≤0.001. SE=Standard error, CRP=C-reactive protein, Hp=Haptoglobin, SAA=Serum amyloid A, APP=Acute phase proteins

## Discussion

SAA has been identified as a valuable or important APP in the diagnosis of inflammation in cattle [16]. Increase in milk SAA levels in cows with mastitis has been reported by Thomas *et al*. [7] and Pyorala *et al*. [17]. Bovine SAA and Hp have been observed to be elevated more in acute rather than chronic inflammatory conditions where α_1_-acid glycoprotein levels are more likely to be elevated. On the other hand, Hp has been recognized as a valuable marker of disease in ruminants where it provides additional information to the traditional hematological investigations [18]. Even Hp has been identified in bronchoalveolar lavage from calves with experimental pasteurellosis [19]. Elevated Hp levels in diseased slaughtered cattle have improved food safety as it has been used extensively due to wider availability of assays for this protein. Significantly high Hp levels were observed in 60 Holstein dairy cattle with lameness due to pododermatitis septic (abscess), pododermatitis circumscripta (sole ulcer), interdigital necrobacillosis, and papillomatous digital dermatitis lesions. In a similar study, significantly higher fibrinogen, Hp and SAA levels in dairy cows suffering from limb diseases as compared to control cows was reported [12]. Other studies have also shown SAA and Hp as the major positive APPs which can increase several folds after tissue injury in cattle [8]. Results consistent with the present study were observed by Nazifi *et al*. [20] with a high concentration of SAA in cows having some inflammatory diseases. SAA concentration in the lame group was about 4.6 times higher than the non-lame group and even 8 times higher in lame animals have *F. necrophorum* positive lameness [14].

**Figure-5:**
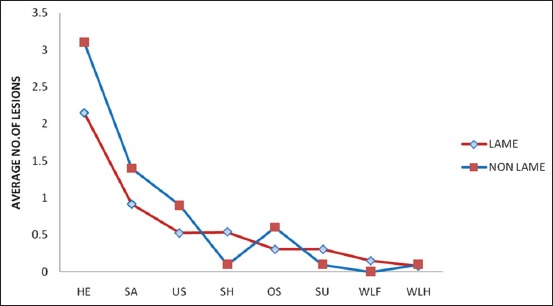
Comparison of average number of lesions in lame and non-lame healthy crossbred cows. (HE: Heel erosions; SA: Sole avulsions; US: Under-run soles; SH: Sole hemorrhages; OS: Overgrown soles; SU: Sole ulcers; WLF: White line fissures; WLH: White line hemorrhage).

Sole ulcers and white line fissures are the lesions responsible for causing clinical lameness in the present study which was further validated by undertaking diagnostic hoof trimming in 13 lame and all the non-lame animals. Average lesion score for various lesions [15] was comparable in lame as well as non-lame animals except there was increased lesion score for sole hemorrhage, sole ulcer, and white line fissures in lame cows (Figure-5). Earlier investigations also recognized sole ulcers, white line disease, interdigital necrobacillosis, and digital dermatitis as the most frequent lesions of the hoof horn and limb skin [21]. In a recent study [13], increased serum Hp concentration in lame cows associated with any of these claw disorders, *viz*., pododermatitis septic (abscess), pododermatitis circumscripta (sole ulcer), interdigital necrobacillosis, and pappilomatous digital dermatitis was recorded. Most of the lesions in the present study were metabolic in origin, and none of the animal was having lesion of infectious origin though Nazifi *et al*. [14] observed comparatively significant acute phase response in lame cattle with interdigital dermatitis as compared to lame cattle with non-infectious lesions.

All the lame cattle in the present study were having SAA concentration above 14 μg/ml; however, Hp levels were approximately 30 times only in 12 animals. This could be because of the fact that Hp is considered usually as a second phase APP in which peak levels are attained at 7-10 days post inflammation [9]. Since blood samples were collected between 3^rd^ and 7^th^ day in the present study that is why peak levels may not have been attained in the lame cows at the time of blood collection.

A 2-3 fold increase in fibrinogen concentration in lame animals was reported by Smith *et al*. [13]. Fibrinogen has been used in cattle as a reliable indicator of bacterial infection, inflammation, or surgical trauma [22]. Similar to Hp, fibrinogen is also a second phase APP whose peak might not be there at the time of sampling in the present study. Abnormal levels of fibrinogen were reported by Smith *et al*. [12] in the majority of treated cows at the time of their return to owners, i.e. mostly after the 6^th^ day of their stay at the clinics. This could be the reason for using Hp and fibrinogen in the post-operative monitoring of infectious complications [22]. Assessing fibrinogen may be more helpful in the post-operative monitoring of infectious complications such as peritonitis because surgical trauma has not been shown to increase fibrinogen concentrations. Plasma fibrinogen concentration was recognized as accurate parameter for differentiating traumatic reticuloperitonitis from other gastrointestinal tract disorders along with Hp levels [22].

Although CRP has been demonstrated in ruminants, however, it is not widely applied in cattle because it is not clear that ruminants’ CRP is an APP. It was proposed that bovine CRP may be lactation-linked rather than synthesized in the liver [23]. A 10-folds increase in CRP levels was reported in mastitis cases [24]. All the lame animals except two in the present study exhibited CRP levels above 2 mg/L indicating its relevance in the diagnosis of inflammatory conditions. The CRP levels in the present study were not comparable with the levels reported by Schrodl *et al*. [24] which could be due to the fact that CRP concentration, being a first phase APP reached at the peak concentration between 1 and 3 days, and levels might have decreased between 3^rd^ and 7^th^ day, the period of sampling. Moreover, CRP has been designated as a moderate (2-10 fold increase) APP in ruminants [8] which is also apparent from the results in the present study.

## Conclusions

It was concluded from the present study that there is a significant increase in Hp, SAA, fibrinogen, and CRP concentrations in the lame crossbred cows as compared to non-lame cows. Lame cows were observed to have comparatively high average lesion score for sole hemorrhages, sole ulcers, and white line fissures. CRP levels though measured for the first time in the lame cattle certainly increased in the lame animals.

## Authors’ Contributions

AB and SSR designed performed the study. AB and SS performed acute phase response assay, whereas AB, SSR, and BKB completed data analysis, revision, and writing of the article. All authors read and approved the final manuscript.
